# Using weedy traits in crops as part of a new green revolution

**DOI:** 10.1111/nph.70224

**Published:** 2025-05-15

**Authors:** Jacob S. Montgomery, Jan E. Leach, Stephen L. Young, Todd A. Gaines

**Affiliations:** ^1^ Department of Agricultural Biology Colorado State University Fort Collins CO 80523 USA; ^2^ Crop Production and Protection, U.S. Department of Agriculture Agricultural Research Service Beltsville MD 20705 USA

**Keywords:** biodiversity, climate‐smart agriculture, genetic diversity, plant abiotic stress tolerance, weed genomics

## Abstract

Crop production faces major challenges, including climate change, biodiversity loss, and global food insecurity, with the need to produce more food under increasingly difficult climatic conditions without negatively impacting ecosystems. Weeds are plants that have adapted to cropping systems despite intensive management efforts over centuries. We propose that weeds possess novel and useful sources of genetic variation that can be used to improve crops for abiotic and biotic stress tolerance. We discuss the potential advantages and disadvantages associated with this approach and outline the interdisciplinary research that will be necessary to successfully identify and utilize this genetic diversity to improve crops. Although the concept of utilizing weedy traits in crops has been put forward previously, recent advances in weed genomics resources, bioinformatics tools to identify the genetic basis of adaptive traits, and genome editing methods now combine to make this approach more feasible.

Throughout the twentieth century, global collaboration led to major technological advancements in agriculture including improvements in crop breeding, management, and biotechnology (Pingali, 2012). This period of innovation is referred to as the ‘green revolution’ and it provides the foundation for modern agriculture. As major challenges threaten to unwind the advances of the green revolution, biotechnology has put a proverbial ‘fork in the road’ for determining the future of agricultural production systems. Using biotechnological tools to alter crop plants and enhance productivity are beneficial, in that they may ultimately improve food availability and reduce agriculture's impact on surrounding ecosystems to a degree that is unachievable through traditional approaches, but these tools are also constrained by ethical and practical considerations. For example, especially stress‐resilient crops with weedy traits developed through biotechnological approaches could escape cultivation and persist/compete in nonagricultural environments, becoming weeds themselves. Additionally, crops improved via biotechnology would need positive public perception to ensure production of crop products that would be desired and utilized by consumers. The complex nature of many crop improvement traits may limit our ability to recapitulate adaptive mechanisms discovered in weeds. However, competitive and persistent crop weeds are genetically diverse, and unlocking their genomic secrets to environmental resilience could identify novel avenues to making crops more resilient. We address such potential difficulties and opportunities on the road ahead as biotechnology utilizing weed genetics in crops becomes increasingly of interest for improving agricultural production systems globally.

Modern agriculture is facing the triple challenge of adapting to climate change, preventing biodiversity loss that occurs when additional land is cleared for crop production and mitigating global food insecurity (Kremen & Merenlender, 2018). Pressure is being felt by growers world‐wide to produce more under increasingly adverse conditions to sustain growing populations without negatively impacting ecosystems (i.e. causing biodiversity loss). Selection for a handful of large‐effect genetic variants in modern crop species during the green revolution led to stepwise improvements in crop production efficiency. However, decades of intensive selection and mining alleles from crops' primary gene pool have driven these large‐effect variants to fixation in modern breeding programs. Today's breeders are focused on the assembly of combinations of many small‐effect variants through strategies like genomic selection in crops. However, weed species may harbor undiscovered adaptive genetic variation that can supplement depleted genetic diversity and further improve crops. While single transgenes have been crucial in providing herbicide, insect, and disease resistance, the full potential of biotechnology has yet to be realized more broadly for abiotic stress tolerance or yield improvement in a majority of crops world‐wide (Khaipho‐Burch *et al*., 2023).

Weeds may function as repositories of adaptive genetic variation for crops. For example, many agricultural weeds have naturally adapted to managed environments, sometimes in their native range, which has allowed them to maintain large effective population sizes (Kreiner *et al*., 2022b) that are genetically diverse (Gaines *et al*., 2021). This is in contrast to most major crops, which have gone through a genetic bottleneck during the artificial selection of traits that are compatible with today's high‐input, monoculture cropping systems, which are often quite different from the environments inhabited by their ancestors and wild relatives. In other examples, weeds have been introduced into an ecosystem and have overcome a genetic bottleneck to rapidly adapt and become established.

Weeds can be tolerant to extreme levels of stress (Fig. 1), and adaptive mechanisms in weeds may be especially applicable for improving crops. Weeds experience selection and thus evolve in agricultural environments alongside crops, so their stress tolerance mechanisms are less likely to be associated with pleiotropic negative fitness effects that would possibly limit their utility. This being the case, any fluctuations in selection pressures faced by weeds should purge alleles that are especially deleterious when selection is relaxed. Additionally, weed species, especially those closely related to crops, with mechanisms of stress tolerance are more likely to utilize pathways that can be effectively transferred or recreated in crop plants while maintaining yield potential.

**Fig. 1:**
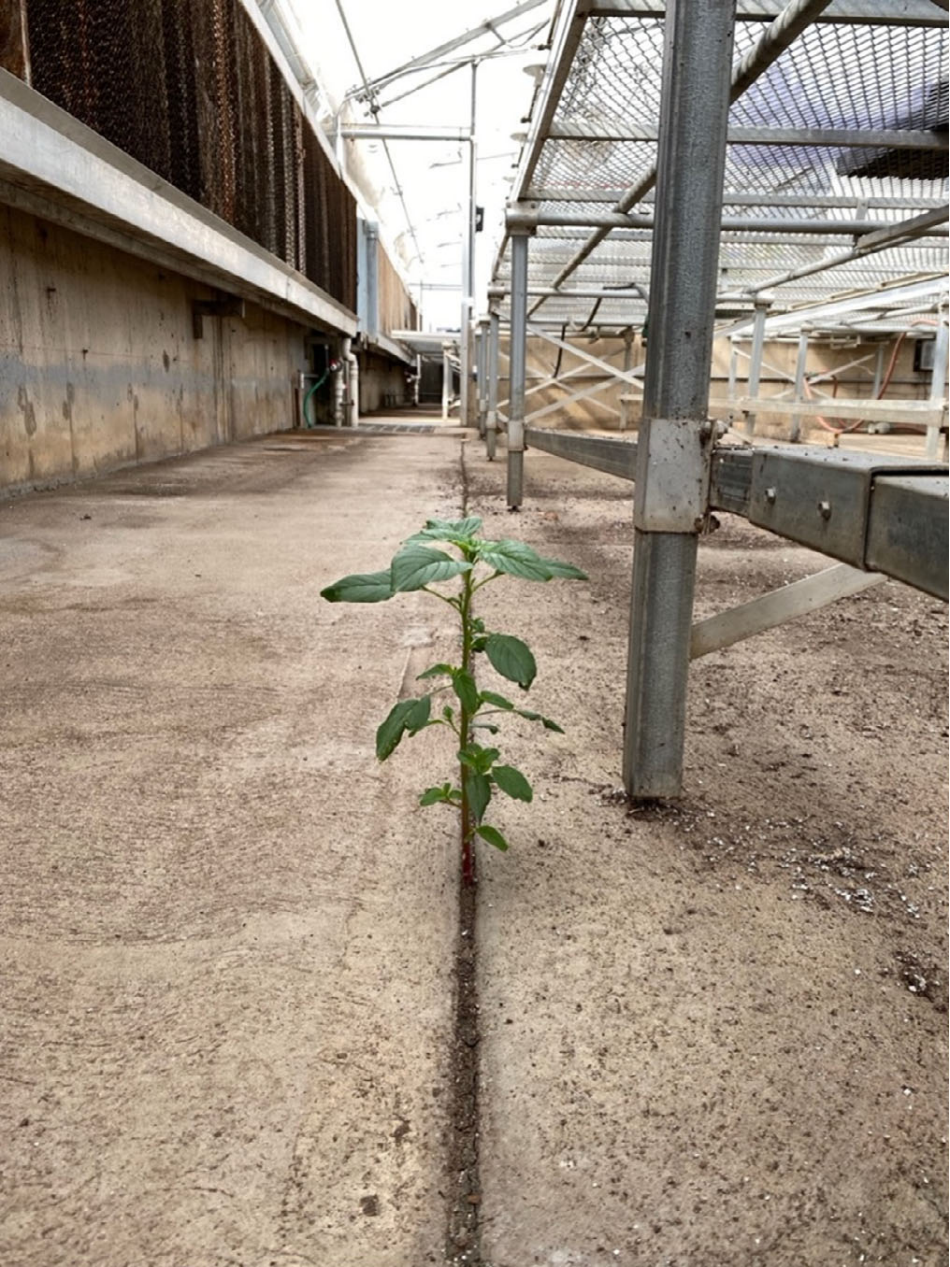
As potential sources of novel abiotic and biotic stress tolerance traits for crop improvement, weeds can survive in extreme environments that are inhospitable to crops. An *Amaranthus palmeri* (Palmer amaranth) plant, a globally prevalent weed, is growing through the cracks of a dry concrete glasshouse floor.

We propose that because crops and weeds are exposed to the same adverse abiotic conditions, their associated biochemical and physiological processes for responding to stress may also be similar. Thus, the ability of weeds to adapt may be translatable to crops more than traits discovered through traditional breeding or synthetic selection efforts in controlled environments. For example, plants must successfully respond to drought duration and intensity for survival (Tuberosa, 2012). This nuance in selection can extend to other abiotic stresses, such as heat, cold, and excessive water (e.g. floods). Further, selection in agricultural settings has likely equipped weeds with biotic tolerance toward pests that also affect crops. The potential biotic stress tolerance traits of weeds toward pathogens or insects could be especially translatable to crop species that have close weedy relatives that are affected by the same pests, such as rice, sorghum, sunflower, and beet. Current gene editing and insertion technologies now allow precise modification of crop genomes, which could soon include adaptive alleles/genes found in weeds (Tonnessen *et al*., 2021).

To date, the focus of most molecular studies on weeds has been to identify and neutralize specific traits that make them difficult to control (e.g. seed shattering, herbicide resistance, or Vavilovian mimicry). Such investigations have successfully identified the genetic basis of qualitative and quantitative traits alike (Ye *et al*., 2019; Gaines *et al*., 2020). However, more recent developments of higher quality genomic references for many diverse weed species (Montgomery *et al*., 2024) and advances in computational approaches (Brixi *et al*., 2025) are providing opportunities to investigate traits aimed specifically at improving crop resilience and productivity (Tonnessen *et al*., 2021). Collection, characterization, and maintenance of diverse germplasm for focal weed species will help facilitate the investigation of such traits, and lessons learned will allow us to reach crop productivity goals like those of the original green revolution.

While the potential for transferring adaptive traits from weeds to crops is enticing, significant barriers will need to be overcome to fully achieve translatable benefits. First, many traits of interest for use in crops are multigenic, complex, and sensitive to environmental conditions, thus making them difficult to isolate and study. Second, the study of adaptive weed alleles for desired traits in crops will require interdisciplinary approaches, possibly including artificial intelligence‐assisted scanning of weed genomes to identify adaptive alleles; generation and/or identification of stress‐sensitive mutants for genetic mapping and/or genome‐wide association analysis; and functional genomic tools to test hypotheses for novel stress tolerance traits. Third, as some weed species are recent invaders, they may arrive preadapted to that environment or evolve to tolerate the new stresses relatively quickly, possibly selecting for a few high‐effect variants suitable as potential targets for biotechnology (Kreiner *et al*., 2022a). However, even if adaptive mechanisms are elucidated in weeds, they may not be translatable to crops because of negative pleiotropic effects. Finally, some weeds have closely related crop relatives, making the application of a new trait potentially more feasible, yet transferring complex traits across more distantly related plant species may prove difficult.

The prospect of mining for alleles in weeds is resourceful and could even be considered a moral obligation in advancing efforts that improve sustainability of agricultural production systems. Being more resourceful and responsible about developing more sustainable agricultural production mirrors, in many ways, how weeds respond to selection based on the environmental conditions they face. It only stands to reason that we should be doing the same. Because of the decades‐long use of genetically similar cultivars for the most common crops in the United States (e.g. corn, soybeans, cotton, wheat), food, feed, and fiber production is becoming increasingly vulnerable to weather extremes and pest outbreaks. Crops need to become more resilient to ensure continued reliable production in current crop geographic distributions and also enable expansion into new locations that currently would be marginal for growing crops. The incorporation of weed traits into crops and their commercialization would lead to a paradigm shift in production agriculture, especially food systems. By first adapting crops in current locations to the changing conditions, land use can remain unchanged, and food security would be improved. Later, as efficiencies improve and a diverse range of crops are more widely adapted, greater selectivity would be available for deciding what to grow and where. In fact, successfully adapted weed species may make good candidates for *de novo* domestication of new crops. For example, *Chenopodium album* is a globally distributed weed species that has been cultivated by some cultures and could be utilized similar to quinoa (*Chenopodium quinoa*) (Correia *et al*., 2024). However, one important consideration in domesticating a weed, as with bolstering current crops, is the possibility of these crops escaping cultivation and de‐domesticating, as has been seen especially for rice (Wu *et al*., 2021). Genetic improvement during domestication may make these new crops into even more tenacious weeds following escape. For example, herbicide‐resistant crops can cause additional management challenges when land use cycles away from that cropping system. This approach may be especially risky if domesticated species are grown in their native range, where outcrossing or escaping cultivation is more likely. Shifting from the production of annual crops to perennial crops, either through changes in cropping systems or through modification of existing annual crops to make them perennial, has been proposed to improve climate resiliency and sustainability (Crews *et al*., 2018). We suggest that utilizing traits from weeds along with *de novo* weed domestication may also be likely to succeed, although all approaches may create new weeds and management issues.

The UN has defined climate‐smart agriculture (CSA) as an approach that attempts to shift the focus from just production and profitability to a greater emphasis on resilience and adaptation to changing environmental conditions. Plant breeders are developing climate‐resilient crops under the CSA framework with enhanced tolerance to weather extremes that maintain or increase yields (Acevedo *et al*., 2020). However, utilizing weed traits in a new climate‐resilient line of crops without yield penalties may far exceed traditional breeding efforts and perhaps even usher in a ‘green revolution 2.0.’. While there is potential of improved crop varieties developing into ‘superweeds,’ this risk pales in comparison to current and projected crop losses due to further havoc as a result of climate change and related natural disasters (Jägermeyr *et al*., 2021).

Climate change is a serious threat and warrants an outside‐the‐box approach for developing climate‐smart agricultural production systems. The use of traits from weed species to produce climate‐resilient crops is a major step in mitigating the effects of climate change and even major pest outbreaks. Weeds are an existing biological source of genetic variation that has been subjected to abiotic and biotic conditions both anthropogenic and natural for centuries and shown to be resilient. The need to advance productivity goals is more urgent than ever, and with collaborations among experts and genomic tools now available, we are equipped to study and eventually commercialize resiliency traits from weeds not possible just a decade or so ago. Ultimately, the transfer of elucidated resiliency traits to crops would bolster stress tolerance and improve food security. This process will require weed scientists, genomicists, physiologists, agronomists, and breeders to work together across geographies at regional to global scales. The creation of such climate‐resilient crops would make production goals achievable in the face of increasing climate instability and world populations.

## Competing interests

None declared.

## Author contributions

JSM, JEL, SLY and TAG were involved in conceptualization; TAG was involved in funding acquisition; JSM, JEL, SLY and TAG were involved in writing – original draft; JSM, JEL, SLY and TAG were involved in writing – review and editing.

## Disclaimer

The New Phytologist Foundation remains neutral with regard to jurisdictional claims in maps and in any institutional affiliations.
